# Development of Skewed Functionality of HIV-1-Specific Cytotoxic CD8^+^ T Cells from Primary to Early Chronic Phase of HIV Infection

**DOI:** 10.1371/journal.pone.0044983

**Published:** 2012-09-13

**Authors:** Wanhai Wang, Chenli Qiu, Chao Qiu, Ying Wang, Xiaoyan Zhang, Jianqing Xu

**Affiliations:** 1 Shanghai Public Health Clinical Center, the Institutes of Biomedical Sciences, Key Laboratory of Medical Molecular Virology of Shanghai Medical College and Institute of Medical Microbiology, Fudan University, Shanghai, China; 2 Shanghai Center for Disease Control and Prevention, Shanghai, China; 3 State Key Laboratory for Infectious Disease Prevention and Control, China CDC, Beijing, China; University of Alabama, United States of America

## Abstract

In recent years, the prevalence of HIV-1 infection has been rapidly increasing among men who have sex with men (MSM). However, it remains unknown how the host immune system responds to the infection in this population. We assessed the quantity of HIV-specific CD8^+^ T-cell responses by using Elispot assay and their functionalities by measuring 5 CD8^+^ T-cell evaluations (IL-2, MIP-1β, CD107a, TNF-α, IFN-γ) with flow cytometry assays among 18 primarily and 37 early chronically HIV-infected MSM. Our results demonstrated that subjects at early chronic phase developed HIV-specific CD8^+^ T-cell responses with higher magnitudes and more diversified functionalities in comparison with those at primary infection. However, populations with IL-2^+^ CD107a^+^ or in combination with other functionality failed to develop in parallel. The multifunctional but not monofunctional HIV-specific CD8^+^ T cells were associated with higher CD4^+^ T -cell counts and lower viral loads. These data revealed that prolonged infection from primary to early chronic infection could selectively increase the functionalities of HIV-specific CD8^+^ T cells in HIV-infected MSM population, the failure to develop IL-2 and cytotoxic functionalities in parallel may explain why the increased HIV-specific CD8^+^ T cells were unable to enhance the containment of HIV-1 replication at the early chronic stage.

## Introduction

The HIV-specific CD8^+^ T-cell responses play an important role in controlling viremia following initial HIV-1 infection, which is supported by several important observations and correlative studies. First, the appearance of cytotoxic CD8^+^ T lymphocytes (CTLs) during acute HIV infection coincided with a decrease in plasma viremia, and the experimental depletion of CD8^+^ T cells *in vivo* resulted in a rapid increase in plasma viremia in the simian immunodeficiency virus infected macaque model [1], [2]. Second, HIV-specific CD8^+^ T-cell mediated immunologic pressure was often manifested by viral escape mutation [3], [4], mutational escape from HIV-specific CD8^+^ T cell responses was associated with progressive increase in HIV plasma viremia [5]. Third, strong correlations were observed between HLA heterogeneity and survival advantage and between certain HLA class I alleles and non-progressive HIV infection [6], for example, HIV-specific CD8^+^T-cell responses restricted by HLA-B*57 were typically associated with a non-progressive clinical consequence or at least a slower disease progression [7], [8].

Though cumulative evidences demonstrated a crucial role for antiviral effects of CD8^+^ T cells, most HIV infected individuals experience progressive loss of CD4^+^ T cells and fail to control plasma viremia despite the presence of vigorous HIV-specific CD8^+^ T-cell responses. A study using the most comprehensive approaches has shown that the magnitude of HIV-specific CD8^+^ T-cell responses may not be a good surrogate for the host capacity to control HIV replication [9]. The role of HIV-specific CD8^+^ T-cell responses in chronic HIV infection may not be appropriately assessed by simply quantifying the level of HIV-specific CD8^+^ T cells. Recent data suggested that the breadth and the magnitude of the CD8^+^ T cell responses directed against HIV-1 as measured by IFN-γ production did not correlate with HIV-1 viral loads [**10]**–[13****]. HIV-1 specific CD8^+^ T cells persisted in high numbers in persons with untreated chronic progressive disease and no quantitative differences in HIV-1 specific T cell responses were observed between individuals with progressive and non-progressive infection [9], [10], [14]. These findings suggested that CD8^+^ T cell characteristics that determine differences in HIV-1 disease outcomes might be of a qualitative rather than a quantitative nature.

CD8^+^ T cells possess multiple functions, including exerting cytolysis through direct interacting with or by releasing cytotoxic molecules to target cells, producing cytokines to inhibit the viral replication, or launching chemokines to block the viral entry into target cells. Therefore, the measurement of one CD8^+^ T-cell function may not provide an adequate evaluation of CD8^+^ T-cell quality. In an effort to better define CD8^+^T-cell quality, a polychromatic flow cytometric assay to simultaneously assess 5 CD8^+^ T-cell functions, including gamma interferon (IFN-γ), tumor necrosis factor alpha (TNF-α), interleukin 2 (IL-2), macrophage inflammatory protein 1β (MIP-1β), and CD107a (a marker of degranulation), has been extensively employed in recent clinical immunological studies and shown that HIV-specific polyfunctional CD8^+^T cells appeared in high number in LTNPs and viral controllers whereas monofunctional HIV-specific CD8^+^ T cells were associated with disease progression and failed to contain viral replication [15].

Recent evidences demonstrated that polyfunctional HIV epitope-specific CD8^+^ T cells were developed during primary HIV-1 infection, but lost their polyfunctionalities and up-regulated programmed death 1 (PD-1) expression in response to persistent viremic infection [16]. However, viral mutational escape consistently occurs during HIV infection and results in the appearance of new epitopes and its-specific CD8^+^T cell responses [17]. Theoretically, the newly developed epitope-specific CD8^+^T cells possess multifunctions, therefore, it could be speculated that a mixture of polyfunctional epitope-specific CD8^+^T cells against newly emerged epitopes with less or even mono-functional epitope-specific CD8^+^T cells targeting at conserved epitopes should be observed during HIV infection. Given HIV infection causes a progressive damage to host immune system and the accumulation of monofunctional CD8^+^ T cells, the consequence of dynamic balance between polyfunctional and monofunctional CD8^+^ T cells is determined by the disease progression and infection time. With the deterioration of host immune system, the polyfunctional CD8^+^ T cells will wane over time and the monofunctional CD8^+^T cells will be likely to overweigh the polyfunctional CD8^+^T cells. Although it is known that the epitope-specific CD8^+^ T cells developed multiple functionalities during primary HIV infection and gradually lose their functionalities with the progression into chronic stages, however, with the addition of the newly developed epitope-specific CD8^+^ T cells with multiple functionalities during chronic infection, the overall pictures of total CD8^+^ T cell responses will be different from the epitope-specific CD8^+^ T cell responses [16], and may more relevant to viral control during HIV-1 chronic infection.

We performed the polychromatic flow cytometric assay as described above in peptide-stimulated peripheral blood CD8^+^ T cells derived from the men who have sex with men (MSM) population, and the functionalities of HIV-specific CD8^+^ T cells were compared between the primarily infected individuals (<6 months) and early chronically infected subjects (12–36 months) to determine the evolution of bulk HIV-specific CD8^+^ T cell functionality at this early stage of HIV-1 infection.

## Results

### Subject Characteristics

The **medians** of blood CD4^+^ T and CD8^+^ T-cell counts, of plasma viral loads, of ages and of infection time for the primary infection (N = 18) and early chronic infection (N = 37) groups were summarized in Table 1. The 18 subjects in HIV primary infection group were infected for a median of 126.5 days (ranged from 47 and to 155 days) whereas those in the chronic phase were infected for 16 months (ranged from 12 to 36 months). Among those subjects in primary infection group, the ages ranged from 21 to 34 years with the median at 26.5 years, CD4^+^ T-cell counts ranged between 94 and 539 cells/µl with the median at 400 cells/µl and 2.70–6.79 log_10_ HIV-1 RNA copies/ml for viral loads with the median at 4.96 log_10_ HIV-1 RNA copies/ml. For thirty-seven subjects in early chronic HIV-1 infected group, the ages ranged between 20 and 52 years old with the median at 30 years old, CD4^+^ T-cell counts ranged 109–848 cells/µl with the median at 366 cells/µl, and viral loads ranged 2.70 to 6.71 log_10_ HIV-1 RNA copies/ml with the median plasma viral load at 4.71 log_10_ HIV-1 RNA copies/ml.

**Table 1:** Clinical characteristics of study subjects.

		Age (years)	CD4 count (cells/µl of blood)	CD8 count (cells/µl of blood)	Plasma viral load (log_10_ RNA copies/ml)	HIV duration a
Primary infection (N = 18)	Medians	26.5	400	836	4.96	126.5 days
	Range	21–34	94–539	306–1383	2.70–6.79	47–155 days
Chronic infection (N = 37)	Medians	30	366	957	4.71	16 months
	Range	20–52	109–848	349–2392	2.70–6.71	12–36 months

a HIV duration refers to the time between HIV diagnosis and sampling date.

### Comparison of HIV-specific IFN-γ-secreting T Cell Responses between Primary and Early Chronic HIV-infected Groups in Response Breadth, Frequency and Magnitude

**Figure 1:**
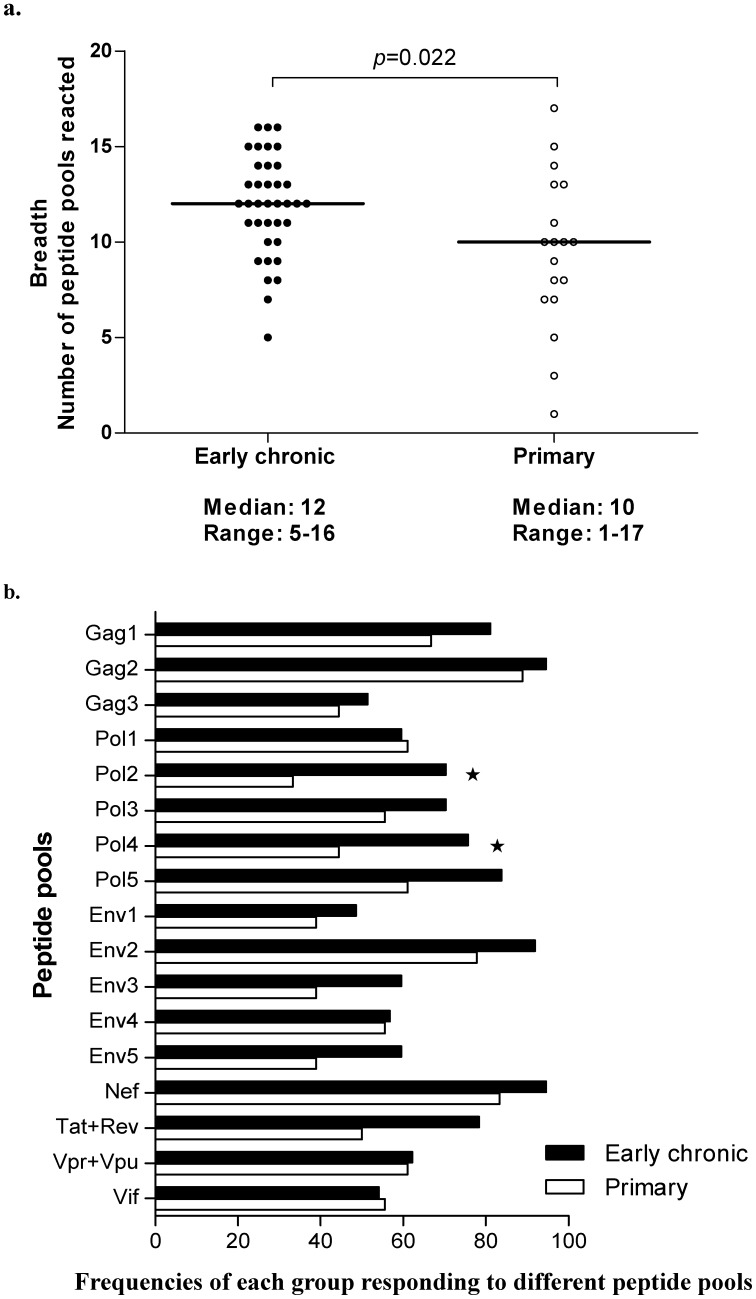
Comparison of the breadth, frequency and magnitude of responses (measured by IFN-γ Elispot and given as SFC/10^6^ PBMCs) between primary and early chronic phase of HIV infection. a. Summary of the number of peptide pools responded (breadth) per individual in primary and early chronic HIV-infected individuals. b. Frequencies of each patient group responding to different peptide pools. The percentages of each patient group mounted a positive response in an Elispot assay to each particular peptide pool were quantified, e.g. greater than 90% of all early chronic subjects mounted a positive response to the Nef pool. Statistically significant differences between groups (p<0.05) were denoted by an asterisk.

An IFN-γ based Elispot assay was employed to quantify HIV-specific CD8^+^ T cells, and the response breadth, the responding frequency and the magnitude of HIV-specific CD8^+^ T cells for each peptide pool were compared between primary infection and early chronic infection groups. As shown in Fig. 1a, the response breadth as defined by the number of peptide pools able to raise positive HIV-specific CD8^+^ T cells was significantly higher in early chronic infection group than the primary infection group (ranged 5–16 peptide pools with the median at 12 for early chronic infection group and 1–17 peptide pools with the median at 10 for the primary infection group, p = 0.022), suggesting that the prolonged HIV-infection could stimulate the host immune system to recognize new antigenic peptide pools and thereby broaden the HIV-specific CD8^+^ T-cell responses. These data were further supported by the observation that the overall responding frequency to 17 peptide pools in early chronic infection group was significantly higher than that in primary infection group (Fig.1b), a dramatic increase in recognition was unambiguously observed for peptide pools of Pol2, Pol4, Pol5, Env3, Env5 and Tat+Rev; Interestingly, only slight increase was observed for the most frequently recognized pools of Gag2, Env2 and Nef with the responding frequencies at 92.7%, 87.3% and 90.9%, respectively, indicating epitopes in those pools are highly immunogenic and able to mount efficient recognition at the primary infection phase (Fig. 1b). Though a trend of increase in the number of HIV-specific CD8^+^ T cells was observed on the majority of peptide pools, a significant increase was mainly identified on those peptide pools with dramatic increase of recognition as described above and Nef peptide pool (p<0.05) (Figure S1). Overall, these data suggested that during the transition from primary infection to early chronic infection, HIV-specific CD8^+^ T cells were expanded in both the breadth and magnitude.

### Functionalities of HIV-specific CD8^+^T Cells During the Primary and Early Chronic Infection

**Figure 2:**
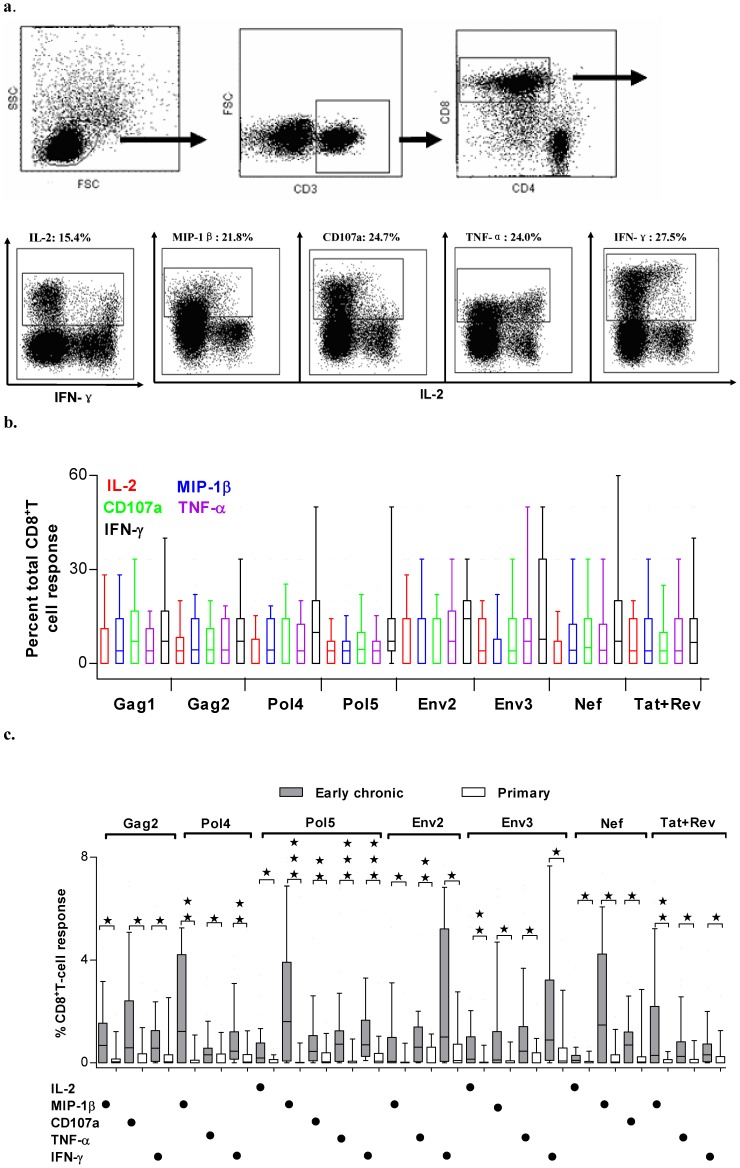
Comparison of the magnitudes of different functional HIV-specific CD8^+^ T cells between primary and early chronic phase of HIV infection. a. Gating scheme used for the identification of CD8^+^ T-cell responses. Data shown were from cells derived from one representative patient, stimulated with PMA and ionomycin. Initial gating was performed on lymphocytes in a forward scatter of FSC-A versus FSC-H, and then FSC vs SSC plot. CD3^+^ events were gated in the FSC versus CD3 plot prior to gating on CD3^+^CD8^+^ and CD3^+^CD4^+^ events. The resulting CD3^+^CD4^-^CD8^+^ population was further gated based on positivity for each of 5 functional responses including IL-2, MIP-1β, CD107a, TNF-α and IFN-γ. b. The frequencies of five distinct functions (color coded as shown) in the total CD8^+^ T-cell response against the indicated HIV peptide mix (x-axis) within 55 HIV-infected MSM. c. Comparison of the magnitude of different functional HIV-specific CD8^+^ T cells between primary and early chronic infection groups. For simplicity, only significantly different populations were shown for Gag1, Gag2, Pol4, Pol5, Env2, Env3, Nef and Tat+Rev responses; no significant differences were identified for those functions not shown. The responses from 55 HIV-infected MSM were standardized so that the profiles could be compared irrespective of any frequency differences. Asterisks were placed above response pairs that were significantly different: ***p<0.001; **p<0.01 and *p<0.05. Each dot denoted a positive response for the function indicated at the bottom left. The box plots represented the 10th, 25th, 75th, and 90th percentiles.

We next determined the functionalities of HIV-specific CD8^+^T cells during primary and early chronic infection. Our data in Fig. 1 have demonstrated that Gag1, Gag2, Pol4, Pol5, Env2, Env3, Nef and Tat+Rev peptide pools are the most predominant peptide pools, therefore, we determined the functionalities of HIV-specific CD8^+^T cells against those peptide pools. A polychromatic flow cytometric assay was employed to simultaneously measure CD107a mobilization, IL-2, MIP-1β, TNF-α, and IFN-γ in CD3^+^CD4^-^CD8^+^ T-cell population. The gating strategy is illustrated in Fig. 2a with data from positive stimulation by PMA plus ionomycin. In this subject, the stimulation of PMA and ionomycin elicited high responses of all five functionalities as IL-2 (15.4%), MIP-1β (21.8%), CD107a (24.7%), TNF-α (24%) and IFN-γ (27.5%). **The magnitude of each function response for each peptide pool was assessed for all study subjects and exemplified in Figure S2,** summed into two groups of primary and early chronic infection and then compared between these two groups. The contribution of each function response toward the total responses against one peptide pool was also calculated and graphed.

As shown in Fig. 2b, all individual function responses for 5 functions were measurable for all 8 peptide pools, IFN-γ producing HIV-specific CD8^+^T cells was the predominant responses in the stimulation of distinct peptide pools whereas IL-2 producing HIV-specific CD8^+^ T cells was the lowest, TNF-α, MIP-1β and CD107a expression were fluctuating between IFN-γ and IL-2 responses. When the immune responses in primary infection group were compared with those in early chronic infection group, we observed increased frequencies of HIV specific CD8^+^ T-cell responses for all five functions in chronic infection in all eight peptide pools (data not shown) and many differences reached statistically significance as shown in Fig. 2c. Several observations should be noticed between these two groups. First, MIP-1β producing HIV-specific CD8^+^ T cells significantly increased from primary to early chronic infection in 7 out of 8 peptide pools and represented the most frequently enhanced responses, and then followed by IFN-γ (6 out of 8 pools), TNF-α (5 of 8 pools), CD107a and IL-2 (both 3 of 8 pools). **These data suggested that different functional responses were not proportionally increased, MIP-1β and IFN-γ might represented the long-lasting functions that could exist in both newly developed HIV-specific CD8^+^ T cells and previously raised CD8^+^ T cells (** **Fig. 2c** **) and thereby the most significant enhancements were manifested in those two functionalities, in contrast, CD107a and IL-2 might only exist in newly developed HIV-specific CD8^+^ T cells and waned in previously activated CD8^+^ T cells.** Second, significant increase in all five functional responses was only observed for Pol5 pool, and then followed by Env3 pool with the increase in four functional responses. **Similar to what we described above in Elispot assay, our ICS data showed that a significant fraction of study subjects recognized these two pools only in the early chronic infection (Figure S3);** thus, a fraction of HIV-specific CD8^+^ T cells against these two pools may represent newly developed immune responses and thereby possess multiple functions. In contrast, less diversified increases were observed in the most immunogenic pools, including Gag2, Env2 and Nef since the majority of HIV-specific CD8^+^T cells have been developed in the primary infection and some may have been undergone functional attenuation. Overall, our flow cytometric data supported our Elispot data and demonstrated that different functional responses were expanded during the transition from primary to early chronic infection, and the development of skewed functionalities of HIV-specific CTLs may have occurred at this early stage.

### Polyfunctional HIV-specific CD8^+^T Cells in Primary and Early Chronic Infection

**Figure 3:**
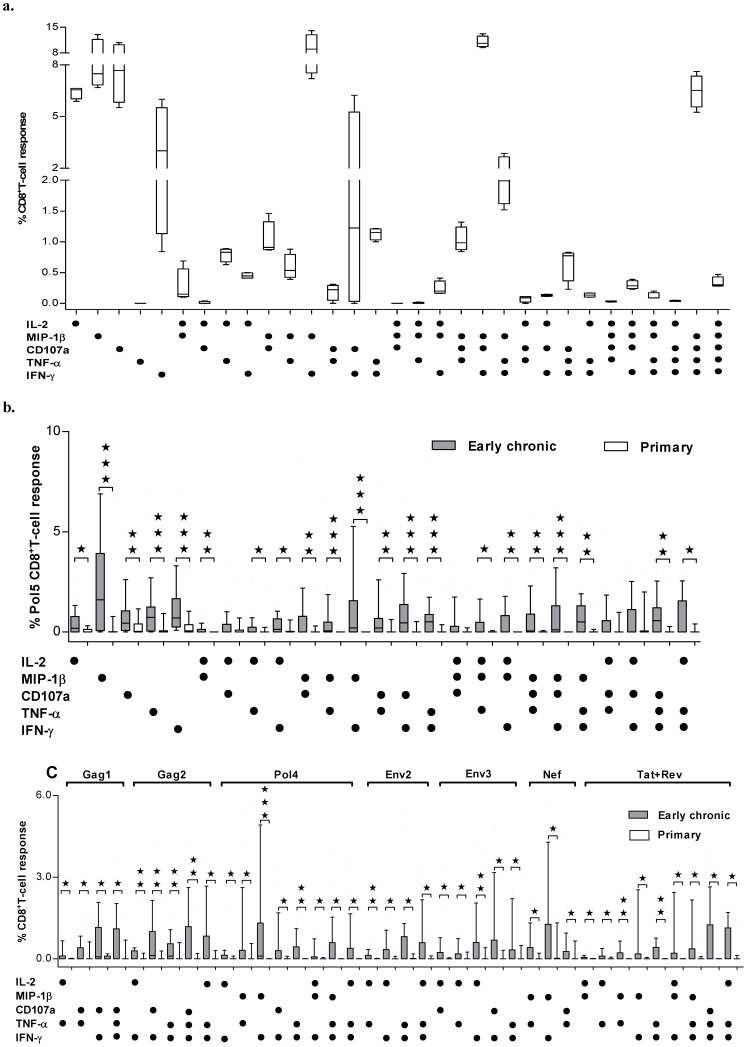
The functional profile of HIV-specific CD8^+^ T-cell populations during primary and early chronic HIV-1 infections. a. Detailed magnitude of different functional CD8^+^ T cells after stimulation with PMA and ionomycin. The bar graph provided fine details on the magnitude of 31 different functional subpopulations; the 10th, 25th, 75th, and 90th percentiles and medians were shown. b. Detailed magnitude of different functional CD8^+^ T cells after stimulation with HIV-Pol5 peptide pool. The bar graph provided fine details on the magnitude of 25 different functional subpopulations (cell subsets with four or five functions were absent in our study); the 10th, 25th, 75th, and 90th percentiles and medians were shown. c. Comparison of the magnitude of different functional HIV-specific CD8^+^ T cells against a certain peptide pool between primary and early chronic infection groups. For simplicity, only significantly different subpopulations were shown for Gag1, Gag2, Pol4, Env2, Env3, Nef and Tat+Rev responses; no significant differences were identified for those functional combinations not shown. In b and c, asterisks were placed above response pairs that were significantly different: ***p<0.001, **p<0.01 and *p<0.05. Each dot denoted a positive response for the function indicated at the bottom left.

On the basis of the expression of each of the five molecules examined, thirty-one different unique subsets of CD8^+^ T cells could be defined, including 5 single functional, 10 dual and 10 triple functional, 5 four functional and 1 five functional subsets. Although the stimulation of PMA and ionomycin does produce CD8^+^ T cells with four or five functions (**Fig. 3a** **and exemplified in Figure S4)**, the cell subsets with four or five functions were not observed in our study subjects after antigenic stimulation, consequently, 25 CD8^+^ T-cell subsets were identified and quantified in our study. To determine the dynamic changes of polyfunctional HIV-specific CD8^+^ T cells, we compared the 25 subsets of HIV-specific CD8^+^ T cells between primary and early chronic infection groups for all 8 peptide pools. As shown in Fig. 3b, 21 out of 25 subsets of HIV-specific CD8^+^ T cells against Pol5 pool were significantly higher in early chronic infection group than that in primary infection group, including eight subsets featured with IFN-γ or MIP-1β producing subsets (MIP-1β^+^, TNF-α^+^, IFN-γ^+^, MIP-1β^+^TNF-α^+^, MIP-1β^+^IFN-γ^+^, TNF-α^+^IFN-γ^+^, CD107a^+^IFN-γ^+^ and MIP-1β^+^CD107a^+^IFN-γ^+^) with the most significantly increase (p<0.001), eight subsets featured with CD107a or IL-2 producing cells (CD107a^+^, IL-2^+^MIP-1β^+^, MIP-1β^+^CD107a^+^, CD107a^+^TNF-α^+^, IL-2^+^MIP-1β^+^IFN-γ^+^, MIP-1β^+^CD107a^+^TNF-α^+^, MIP-1β^+^TNF-α^+^IFN-γ^+^, CD107a^+^TNF-α^+^IFN-γ^+^) with p<0.01 and five subsets producing IL-2 or in combination with other cytokines (IL-2^+^, IL-2^+^TNF-α^+^, IL-2^+^IFN-γ^+^, IL-2^+^MIP-1β^+^TNF-α^+^ and IL-2^+^TNF-α^+^IFN-γ^+^) with p<0.05. When HIV-specific CD8^+^ T cells against other 7 pools were examined, several observations should be noticed (Fig. 3c). First, different increasing pattern of HIV-specific CD8^+^ T cell subsets were observed among different pools, diversified subsets were increased for peptide pools with significantly increased recognition during early chronic infection from primary infection, such as Pol4, Pol5 and Tat+Rev pools, and less subsets were increased for immunogenic pools, including Gag2, Env2 and Nef pools. Second, only multifunctional subsets are significantly increased from primary infection to early chronic infection, including 9 dual functional and 1 triple functional subsets (p<0.01). Among all increased populations against those 7 pools, 14 out of 38 subsets are TNF-α^+^IFN-γ^+^ or TNF-α^+^IFN-γ^+^ in combination with other functions, 8 subsets are MIP-1β^+^TNF-α^+^, MIP-1β^+^IFN-γ^+^ or MIP-1β^+^IFN-γ^+^ in combination with other functions, 16 subsets are IL-2^+^ or CD107a^+^ in combination with other functions. However, IL-2^+^CD107a^+^ subset or IL-2^+^CD107a^+^ in combination with any one of IFN-γ, MIP-1β and TNF-α, only showed slight increase in early chronic infection group and did not reach statistical significance with only one exception in Env3 pool in which IL-2^+^CD107a^+^ subset reached statistical significance but the magnitude was relatively low comparing to other subset responses.

**Figure 4:**
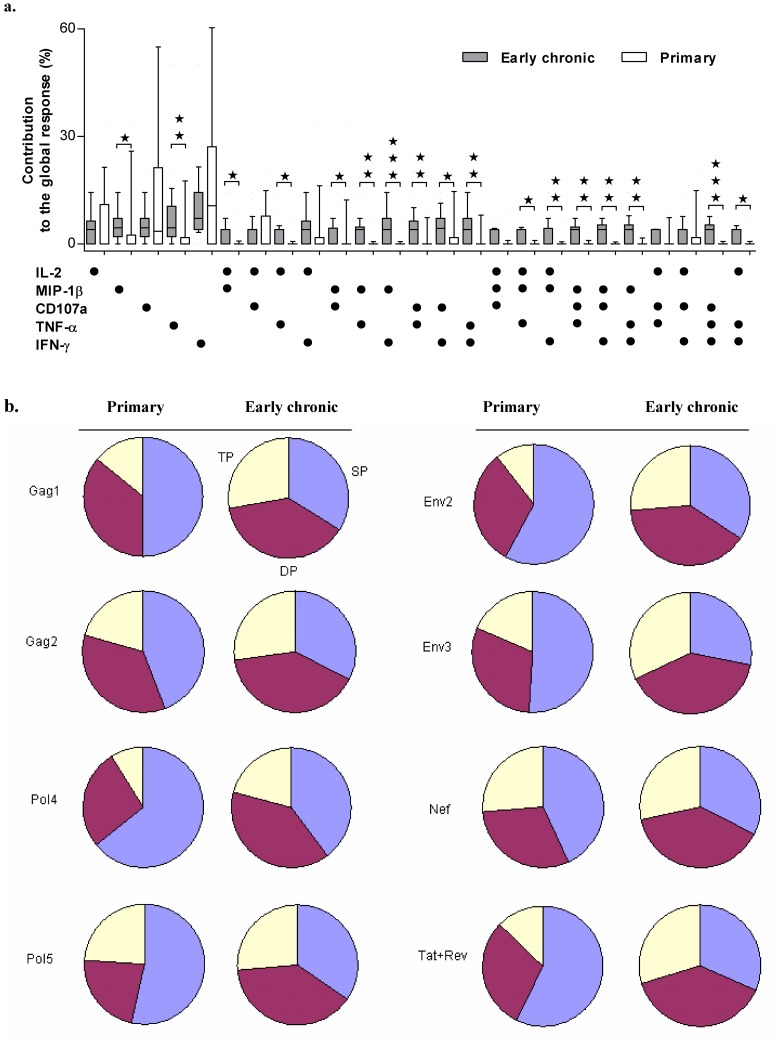
Comparison of the weighted contribution of different functional CD8^+^ T-cell subsets to the global HIV peptide pool-specific CD8^+^T-cell responses between primary and early chronic HIV-1 infection groups. a. Detailed weighted contribution of different functional CD8^+^T subsets to the global CD8^+^ T response against HIV-Pol5 peptide pool. HIV-specific CD8^+^ T cells from early chronic infections have a qualitatively different functional profile compared to primary infections. The box plots represent the 10th, 25th, 75th, and 90th percentiles of the proportion of the respective functional responses toward the total CD8^+^ T-cell responses. Asterisks were placed above response pairs that were significantly different: ***p<0.001; **p<0.01 and *p<0.05. Each dot denoted a positive response for the function indicated at the bottom left. b. Proportions of triple producers (TP), double producers (DP) and single producers (SP) of CD8^+^ T cells responding to eight peptide pools during primary and early chronic HIV-1 infections, were shown in the pie charts, respectively.

We also determined the composition of HIV-specific CD8^+^ T cell immune responses by calculating the proportion of each functional subset in global peptide pool-specific responses and compared them between primary and early chronic infection groups (Fig. 4a and 4b). As shown in Fig. 4a, the majority of Pol5-specific CD8^+^ T cell immune responses in primary infection were single functional and less functionally diversified, and both dual- and triple-functional CD8^+^ T cells were significantly increased in the early chronic infection group. This observation also held true for all other 7 peptide pools (Fig. 4b).

**Figure 5:**
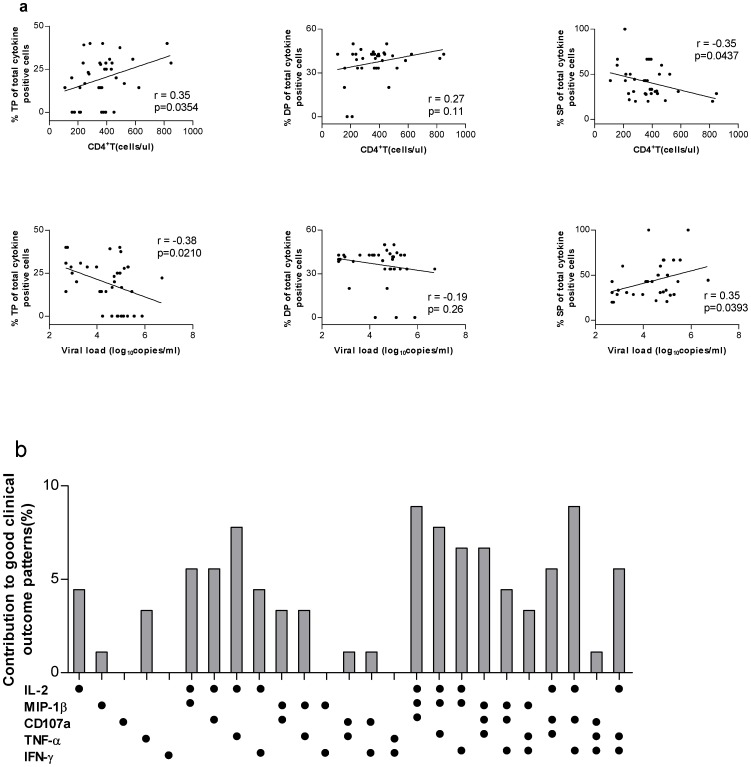
Correlations between CD8^+^ T-cell functionalities and blood CD4^+^ T-cell counts or plasma viral loads during early chronic HIV-1 infections. a. Correlation between the CD8^+^ T-cell functionalities and blood CD4^+^T-cell counts or plasma viral load for triple producers (TP), double producers (DP), and single producers (SP) of Gag2-specific responses in early chronic HIV-1-infected individuals. b. Different functional HIV-specific CD8^+^ T cells associated with good clinical outcomes (good clinical outcomes indicated an inverse correlation between the magnitude of HIV-specific CD8^+^ T-cell responses and viral loads, or a positive correlation between the magnitude of HIV-specific CD8^+^ T-cell responses and CD4^+^ T-cell counts). Results from linear regression model analysis on the magnitude of eight HIV-peptide pool specific responses against blood CD4^+^ T-cell counts or plasma viral loads during early chronic HIV-1 infections. Each dot denoted a positive response for the function indicated at the bottom left.

Overall, our data demonstrated that the transition from primary infection to early chronic infection resulted in the increase of polyfunctional T cell subsets, including dual- and triple-functional CD8^+^ T cell subsets; different functional subsets are dis-proportionally increased, IL-2 and CD107a positive T cell subsets are less increased comparing to INF-γ or MIP-1β producing T cell subsets, which may implicate that IL-2 and CD107a producing T cell subsets are more sensitive to viral persistence. In addition, the prolonged infection could result in the increase of functionality for less immunogenic peptide pools.

### The Association between Polyfunctional CD8^+^T Cells and CD4^+^T-cell Counts, Plasma HIV-RNA, and Clinical Outcomes

Previous studies have demonstrated a strong association between the maintenance of polyfunctional HIV-specific CD8^+^T cells and the containment of HIV-1 replication or disease progression [15], [18]. Here, we evaluate the functionality of HIV-specific CD8^+^T cells and determined its association with the disease progression represented by CD4^+^T cell counts and viral replication expressed in viral loads. Since Gag2 peptide pool raised HIV-1 specific CD8^+^T cell responses in 92.7% subjects in our cohort and represented the most predominant HIV-1 specific CD8^+^ T cell responses, it is reasonable to use Gag2-specific CD8^+^ T cell immune responses to define the association first. As shown in Fig. 5a, an inverse association between the single functional T cell responses and CD4^+^ T -cell counts (r = -0.35, p = 0.04) was observed, in contrast, positive associations were identified between the triple functional T cell responses and CD4^+^ T -cell counts (r = 0.35, p = 0.03) and between the dual functional T cell responses and CD4^+^ T-cell counts though the statistical significance was not reached. In accordance with these observations above, the increase in the single functional T cell responses was coincided with the increase of plasma viral loads (r = 0.35, p = 0.04) and an inverse association was observed between the triple functional T cell responses and plasma viral loads (r = −0.38, p = 0.02) and between the dual functional T cell responses and plasma viral loads though the statistical significance was not reached. In addition, by using Spearman rank correlation, we also analyzed the associations between other seven HIV-peptide pools specific CD8^+^ T -cell responses with CD4^+^T-cell counts or plasma viral loads and similar patterns of associations were observed (data not show). Overall, our data indicated that the polyfunctional HIV-specific CD8^+^T cells **were correlated to slow disease progression and low viral replications** whereas the single functional HIV-specific CD8^+^T cells failed to do so.

We further analyzed the contribution from each subset to the good clinical outcomes by employing linear regression model analysis. **Good clinical outcomes were defined as an inverse correlation between the magnitude of HIV-specific CD8^+^ T-cell responses and viral loads, or a positive correlation between the magnitude of HIV-specific CD8^+^ T-cell responses and CD4^+^ T-cell counts**. As shown in Fig. 5b, a contribution rank of 3-functional subsets >2-functional subsets >1-functional subsets was observed. All three functional subsets could contribute to the good clinical outcomes with the highest contributions from two subsets of IL-2^+^CD107a^+^MIP-1β^+^ and IL-2^+^CD107a^+^IFN-γ^+^ (8.89% for both), then followed by other IL-2 or CD107a positive subsets. For dual functional subsets, IL-2 in combination with other function ranked as the highest contributor, then followed by subsets of CD107a in combination with other functions. These data indicated that IL-2 producing and/or CD107a positive T cell subsets contributed the most to the good clinical outcomes and thereby represent the most powerful T cell subsets to contain viral replication and restrain the disease progression.

## Discussion

In this study, we examined and compared HIV-specific CD8^+^T-cell functional hierarchy between HIV-1 primarily infected (<6 months) and early chronically infected MSM subjects (12-36 months) by **using peptide derived from consensus B clade**. Our results suggested that the prolonged HIV-1 infection as shown in early chronic phase group stimulated a broader HIV-specific CD8^+^T cells with a higher magnitude and more diversified functionalities. The kinetic profile of HIV-specific CD8^+^ T cells varied among the immunogenic and less immunogenic peptide pools, the prolonged stimulation is required for less immunogenic epitopes to develop more functionally diversified and vigorous HIV-specific CD8^+^T cells whereas less kinetic changes were observed for immunogenic peptide pools. In addition, the polyfunctional HIV-specific CD8^+^T cells were associated with higher CD4^+^ T-cell counts and lower viral loads whereas the single functional CD8^+^T cells were coincided with lower CD4^+^T-cell counts and higher viral loads, indicating that different functional HIV-specific CD8^+^ T cells are entitled with varied capacity to control viral replication.

These data are for the first time to detail the overall functional kinetic profiles of HIV-specific CD8^+^T cells in primarily and early chronically HIV-infected MSM subjects and identify the changes of HIV-specific CD8^+^T cells in functionalities, magnitude and breadth during this transitional phase. In accordance with previous reports [16], our data demonstrated that HIV infection elicited polyfunctional HIV-specific CD8^+^T cells in early phase. However, prolonged HIV stimulation is a double-edged sword, on one hand, it could increase the total number of polyfunctional CD8^+^ T-cells and thereby may improve the control of viral replication; on the other hand, it may lead to the functional damage to HIV-specific CD8^+^ T cells due to the viral persistent stimulation. Our data suggested that IL-2 producing function was less developed and may have been partially damaged even in the early chronic infection phase, this observation is conciliated with previous observations that IL-2 producing subsets are mainly appeared in early HIV-1 infection and in the relative healthy immune environment of LTNPs [15], [16], [18]-[22]. Interestingly, the function of degranulation (CD107a) seemed also to be partially damaged in the early chronic infection phase in our cohort, which could be resulted from two aspects. First, the persistent HIV infection caused the continued degranulation of HIV-specific CD8^+^ T cells, cytotoxic molecule producing subsets are usually effector cells and less capable of proliferation [23], thereby prone to be exhausted. Second, the gain of cytotoxic activities during the differentiation of HIV-specific memory CD8^+^ T cells may depend on the help of CD4^+^ T cells [24], which is waned over time during HIV infection and thereby less cytotoxic molecule producing cells are differentiated from HIV-specific memory CD8^+^ T cells with prolonged HIV infection. Overall, the degranulation function is less increased and the capacity for HIV-specific CD8^+^T cells to kill HIV-infected target cells is blunted even in the early stage. Therefore, the viral suppression during early phase of HIV infection is depended on the balance between the gain of polyfunctional HIV-specific CD8^+^T cells which could enhance the capacity to contain the viral replication and the loss of cytotoxic activities and proliferative capacity which attenuate the ability to eliminate HIV-infected target cells, and with the elapse of infection time this balance will progressively incline to the loss of functions [25], [26]. In addition, although total multiple functional CD8^+^ T cells are increased from primary infection to the early chronic infection, the accumulation of mutational viruses at this stage resulted in the increase of specificities of epitopes and thereby may reduced the multiple functional CD8^+^ T cells per epitope, therefore, it is not surprised that the viral control from primary infection to early chronic infection was not significantly improved.

Indeed, HIV infection not only causes the shift of the functionalities of HIV-specific CD8^+^ T cells as shown in our study above and in previous reports [16], the prolonged HIV infection also results in the shift of the specificities of targeted epitopes recognized by HIV-specific CD8^+^ T cells [27], [28]. As known, immune escaping mutation could occur within two weeks after HIV infection and continued to do so thereafter [29], [30]. The immune escape from the attack by dominant T cell responses could cause the exposure of subdominant epitopes to immune system and thereby stimulates new HIV-specific CD8^+^ T cells against those subdominant epitopes [17], [28], [31], [32], which practically results in the increase in both the breadth and the magnitude of HIV-specific CD8^+^T cells as observed in our study. Since the hierarchy of epitope immunogenicity determines the timing for activation during HIV infection and the immunogenicity varied among different HIV epitopes/antigens, HIV-specific CD8^+^ T cells against different epitopes/antigens could be developed at different time, and immunogenic epitopes, usually designated as dominant epitopes, could raise CD8^+^T-cell responses earlier than that less immunogenic epitopes (subdominant epitopes) [17], [27], [28], [31]. This governing rule for CD8^+^ T-cell activation determines that antigen/peptide pools containing dominant epitopes will mount good immune responses in primary infection and a significant further increase during prolonged infection will be unlikely to occur, which is proven by the observation in this study for Gag2 and Nef peptide pools. In contrast, antigen/peptide pools containing subdominant epitopes will be likely to raise new waves of immune responses during prolonged infection and thereby a significant increase could be observed after primary infection, as shown for peptide pools of Pol2, Pol4, Pol5, Env3, Env5 and Tat+Rev in our study. However, the sequential activation of CD8^+^T-cell responses against HIV infection could cause problem and render HIV-1 avoid the simultaneous attack from both immunogenic and less immunogenic epitope specific T cell responses, and thereby allow the virus to implement a sequential escaping strategy. This observation has important implication for HIV-1 vaccine development, particularly a vaccine strategy to simultaneously raise vigorous CD8^+^ T cells against both immunogenic and less immunogenicity will be likely more effective.

The functionality of subdominant epitope specific CD8^+^T cells could be dramatically different from dominant epitope specific CD8^+^T cells, because the lower avidity of subdominant epitope with its corresponding T-cell receptors may impart CD8^+^ T cells with discern intrinsic difference from dominant epitope [33]. In addition, the reduced help from CD4^+^ T cells may also contribute to the functional difference [25]. Though previous studies demonstrated that CD8^+^ T cell specificity is one of critical determinants in viral suppression [1]-[5], [34], it remains to be addressed whether this was attributed to the different functionalities for different CD8^+^T-cell specificity or to the varied mutational damage to viral fitness for different CD8^+^ T-cell specificity or to both. In summary, HIV-specific CD8^+^T-cell specificities are continuously expanded over time by engaging in additional less immunogenic epitopes, it remains to determine whether the functional features of CD8^+^ T-cell specificity will influence their capacity to suppress viral replication.

Polyfunctional HIV-specific CD8^+^ T cells have been associated with good clinical outcomes [15], [18]. The reason for this is that polyfunctional T cells are fully armed and could use multiple tools to tackle with HIV, and thereby more potent to suppress HIV replication. In accordance with those observations, our data corroborated that triple functional CD8^+^ T cells were significantly correlated with higher CD4^+^T-cell counts and lower viral loads whereas single functional CD8^+^T cells were unable to do so. Furthermore, our data emphasized that IL-2 and CD107a dual positive CD8^+^ T cells were among the most powerful T cells to contain HIV replication. **Since IL-2** ^+^ **CD8** ^+^ **T cells have the potential to proliferate independent of CD4** ^+^ **T helper** [**35**] **, we speculated that although HIV infection resulted in the depletion of CD4** ^+^ **T cells, IL-2** ^+^ **CD107a** ^+^ **CD8** ^+^ **T cells could proliferate despite the absence of help from CD4** ^+^ **T cells and thereby increase the number of HIV-specific CD8** ^+^ **T cells, and enhanced the capacity to contain the viral replication.**

Unexpectedly, we failed to identify HIV-specific CD8^+^ T cell subsets with four and five functions which had been frequently reported for LTNPs [15]. This is highly unlikely to be caused by technical problems since all five functions are well defined in less three functional subsets and the stimulation of PMA and ionomycin does produce the CD8^+^ T cells with four or five functions. It is unknown whether the limited functionalities are MSM population specific or simply a further prolonged stimulation is required for four and five functional T-cell subsets, additional cohort studies should be performed to address this question. **In addition, both B clade and non-B clade HIV viruses have been circulating in MSM populations** [**36**] **, the usage of consensus B derived peptides in our assay quantified the cross-reactive CD8^+^ T cells and thereby was likely to underestimate the magnitude of CD8^+^ T cells responses, it remains unknown whether the differences observed in this study will be applicable to autologous CD8^+^ T cell responses.**

## Materials and Methods

### Study Subjects

A cross-sectional study was conducted on 55 ART naïve HIV-1-positive MSM subjects. Eighteen individuals with HIV-1 primary infection, defined as HIV-1 seroconversion within 6 months prior to study enrollment, and thirty-seven subjects with early chronic infection, defined as HIV-1 seroconversion for 12-36 months, were recruited at Chaoyang CDC, Beijing, China. The study protocol was evaluated and approved by both institutional review boards at Shanghai Public Health Clinical Center and at the Chaoyang CDC in Beijing, and all individuals provided written informed consent for participation in the study.

### Measurement of CD4^+^ T-cell Counts and Quantification of Plasma Virus RNA

CD4^+^ T-cell counts and HIV-1 plasma virus RNA were performed as described previously [37]. TruCount™ tubes and TriTEST™ reagents (CD3-FITC/CD4-PE/CD45-PerCP) were purchased from BD Biosciences for counting CD4+ T cells, all procedures were followed as suggested by the manual. Real-time RT-PCR was used to determine plasma viral loads by amplification of a 102-bp segment of the HIV-1 *gag* gene. Briefly, Real-time RT-PCR was performed using Real-time RT-PCR Kit (**S**henzhen PG Biotech Co. China) in 10 µl reaction volume, reactions were performed in triplicate by using a COBAS AMPLICOR Analyzer. Results were expressed as HIV-1 RNA copies/ml. The low limitation was 500 copies/ml.

### HIV Peptides and Antibodies

HIV-1 clade B consensus peptides obtained from NIH AIDS Research and Reference Reagent Program, spanning the entire HIV proteome. These peptides were 15-mers overlapping by 11 residues and were specific to consensus sequences for HIV-1 clade B Gag (123 peptides, cat. no.8117), Pol (249 peptides, cat. no. 6208), Env (211 peptides, cat. no. 9480), Tat (23 peptides, cat. no.5138), Rev (27 peptides, cat. no. 6445), Nef (49 peptides, cat no. 5189), Vif (46 peptides, cat no. 6446), Vpr (22 peptides, cat no. 6447), and Vpu (19 peptides, cat no. 6444). All peptides were synthesized as free acids and were more than 80% pure. Individual lyophilized peptides were resolved into 50 µl DMSO (Sigma) and 950 µl RPMI 1640 at 1 mg/ml for the subsequent formulation of peptide pools. Seventeen peptide pools were formulated, including five pools for Pol (Pol1, Pol2, Pol3, Pol4, and Pol5; each contains 50 peptides except Pol5 contains 49 peptides) and for Env (Env1, Env2, Env3, Env4, and Env5; each contains 42 peptides except Env 5 contains 43 peptides), three for Gag (Gag1, Gag2, and Gag3; each contains 41 peptides), one for Tat and Rev peptides (Tat+Rev), one for Vpr and Vpu peptides (Vpr/u), one for Nef and one for Vif. All peptide pools were stored at −80°C and the final concentration for each peptide in the cultures was 2 µg/ml.

Directly conjugated antibodies for flow cytometric analysis were used in the following fluorochrome combinations available from commercial sources, including CD8-AmCyan, IL-2–phycoerythrin (PE), CD107a-Cy5-PE, TNF-α-allophycocyanin (APC), CD4-Cy7-APC from BD Biosciences, CD3-ECD from Beckman Coulter, IFN-γ-Pacific Blue from eBioscience, MIP-1β-fluorescein isothiocyanate (FITC) from R&D Systems. The unlabeled costimulatory antibodies to CD28 (clone L293) and CD49d (clone L25), were purchased from BD Biosciences.

### Elispot Assay

Fresh PBMCs were used in an interferon (IFN)-γ based Elispot assay as described previously [37]. The number of specific T cells was counted with ChampSpot IV Bioreader (Beijing SAGE Creation Science, Beijing, China). The number of spot forming cells (SFCs) was defined by subtracting the negative control values (background) and the average number of spots in duplicate wells per 10^6^ PBMCs. The background was <15 SFCs/10^6^ PBMCs (3 spots/well at 200,000 PBMCs/well) in all cases and responses ≥50 IFN-γ SFCs/10^6^ PBMCs and **>3 times of background** were considered as positive responses.

### Intracellular Cytokine Staining (ICS) Assay

All intracellular cytokine staining (ICS) assays were carried out on freshly isolated PBMCs. Purified PBMCs were resuspended at 1×10^7^ cells/ml in complete RPMI 1640 medium and were then aliquoted (1×10^6^ cells) at 200 µl to each well in the 96-well U-bottom plates (Costar Inc., NY). PBMCs were stimulated with 8 pools of overlapping peptides (2 µg/ml final concentration for each individual constituent peptide final concentration) spanning expressed HIV-1 gene products (Gag1, Gag2, Pol4, Pol5, Env2, Env3, Nef, and a combined pool of Tat and Rev), and costimulatory antibodies (anti-CD28 and anti-CD49d at a final concentration of 1 µg/ml each) and CD107a-Cy5-PE (pre-titered volume) were added to the cells. Cells were incubated for 6 hours at 37°C in the presence of 5% CO2, and the secretion inhibitor brefeldin A (final concentration of 10 µg/ml, Sigma) was added to the cultures from the second hour of incubation. A negative control (peptide-free medium plus 0.05% DMSO and costimulatory antibodies) and a positive control (stimulated with PMA and ionomycin at a final concentration of 50 ng/ml and 1 µg/ml, respectively; Sigma) were included in each assay.

Following incubation, the cells were washed with phosphate-buffered saline (PBS)-2% FBS and stained with surface antibodies (CD3-ECD, CD4-Cy7-APC and CD8-AmCyan) for 30 min at 4°C in the dark. Cells were then washed again, fixed and permeabilized using the Cytofix/Cytoperm kit (BD PharMingen) according to instructions. Following fixation, the cells were washed twice in the perm buffer and stained with antibodies against intracellular markers. Cells were then incubated for 30 min at 4°C with a cocktail of four anti-human monoclonal antibodies conjugated to different fluorochromes for the detection of cytokines (IL-2-PE, MIP-1β-FITC, TNF-α-APC and IFN-γ-Pacific Blue). Following staining, the cells were washed, fixed (PBS containing 1% paraformaldehyde), and stored at 4°C until analysis (within 24 h) in a modified FACSAria™ flow cytometer (BD Immunocytometry System).

### Flow Cytometry Analyses

Data acquisition and analysis was performed using the BD FACSDiva software. Instrument settings and fluorescence compensation were performed for each day of testing by using unstained and single fluorochromes-stained samples. Stimulated cells stained with surface molecules and isotype controls corresponding to intracellular markers were included for each patient in order to accurately set negative populations. Lymphocyte gating was performed on small cells in a forward scatter (FSC) versus side scatter (SSC) plot. Between 200,000 and 500,000 events were acquired in the lymphocyte gate. CD3^+^ events were gated in an FSC versus CD3 plot prior to gating on CD4 versus CD8, and CD3^+^CD4^-^CD8^+^ cells were considered to be CD8^+^T cells. Following identification of CD8^+^T cells, individual gating was performed for each studied function and their combinations. For this purpose, “derived gating tools” available at the FACSDiva software was used. To study double- and triple-positive populations (cell subsets with four or five functions were practically absent), intersections of two and three gates were created, respectively. Data presented correspond to background-subtracted results by using the anti-CD28/49d stimulation alone as background. This was performed on a cytokine subset by cytokine subset basis, i.e., subtracting the result from the “CD28/49d-only” for a given cytokine subset to the same subset of a peptide-stimulated condition.

### Statistical Analysis

Unless otherwise noted, net antigen-specific responses were calculated by subtracting values for the negative control (medium plus costimulatory antibodies) from antigen-specific responses. A positive response threshold was set at 50 SFCs/10^6^ PBMCs and 0.05% of CD8^+^ T cells in the Elispot assay and ICS assay, respectively. In ICS assay, values below this level, after subtraction of negative control values, were set to zero [15]. Statistical analyses were performed using GraphPad Prism 5 for Windows (GraphPad Software, La Jolla, CA). All data were analyzed using Mann–Whitney U tests of nonparametric statistics. Correlations were determined using Spearman’s rank test. All tests were considered significant if the *p* value obtained was less than 0.05.

## Supporting Information

Figure S1 Comparison of the magnitude of CD8^+^ T cell responses to each peptide pools per individual between groups. Elispot assays were used to determine the magnitude of CD8^+^ T cell responses. Filled circles were used for early chronic HIV-infected subjects and open circles for primary HIV-infected subjects; The SFCs from control settings were deducted from experimental setting and therefore the spots shown in this figure were “net” SFCs for comparison. Statistically significant differences between groups (p<0.05) were denoted by a line with an asterisk, and p<0.01 for double asterisks. The median magnitude for each group are indicated as horizontal black lines. The p value was calculated by using a nonparametric Mann–Whitney test.
(TIFF)

Figure S2 Gating scheme used for the identification of CD8^+^ T-cell responses. Data shown were from cells derived from one representative patient, stimulated with Gag 1 peptide pool. Initial gating was performed on lymphocytes in a forward scatter of FSC-A versus FSC-H, and then FSC vs SSC plot. CD3^+^ events were gated in the FSC versus CD3 plot prior to gating on CD3^+^CD8^+^ and CD3^+^CD4^+^ events. The resulting CD3^+^CD4^-^CD8^+^ population was further gated based on positivity for each of 5 functional responses including IL-2, MIP-1β, CD107a, TNF-α and IFN-γ.
(TIFF)

Figure S3 Comparison of the magnitude of CD8^+^ T cell responses to each peptide pools between groups. Intracellular staining assays were used to determine the magnitude of CD8^+^ T cell responses. Statistically significant differences between groups (p<0.05) were denoted by a line with an asterisk, and p<0.01 for double asterisks and p<0.001 for three asterisks. The median magnitudes for each group were indicated as horizontal black lines. The p value was calculated by using a nonparametric Mann–Whitney test.
(TIFF)

Figure S4 Gating scheme used for the identification of different functional CD8^+^ T-cell subpopulations. Data shown were from one representative patient, stimulated with PMA plus Ionomycin (positive control). Cells collected from diagonal line in FSC-A vs FSC-H plot were used for subsequent analysis, both CD3 and CD8 surface markers were employed to determine CD8^+^ T-cell population. Different functional CD8^+^ T cells were determined as followings: IFN-γ was firstly tested, and then both IFN-γ positive and negative cells were further determined on the plot of TNF-alpha vs MIP-1belta, finally all four populations from the plot of TNF-alpha vs MIP-1belta were tested on the plot of IL-2 vs CD107a. The percentage for each functional CD8^+^ T cell population was calculated against CD8^+^ T cells, eg. IFN-γ^+^TNF-a^+^MIP-1β^+^CD107a^+^IL-2^-^ population is calculated by 13.9%×5.8%×54.6% = 0.44%.
(TIFF)
