# Supplementary figures and images for: Development of Skewed Functionality of HIV-1-Specific Cytotoxic CD8+ T Cells from Primary to Early Chronic Phase of HIV Infection
Source: PLoS One. 2012 Sep 13;7(9):e44983. doi: 10.1371/journal.pone.0044983 (PMC3441698; doi:10.1371/journal.pone.0044983)

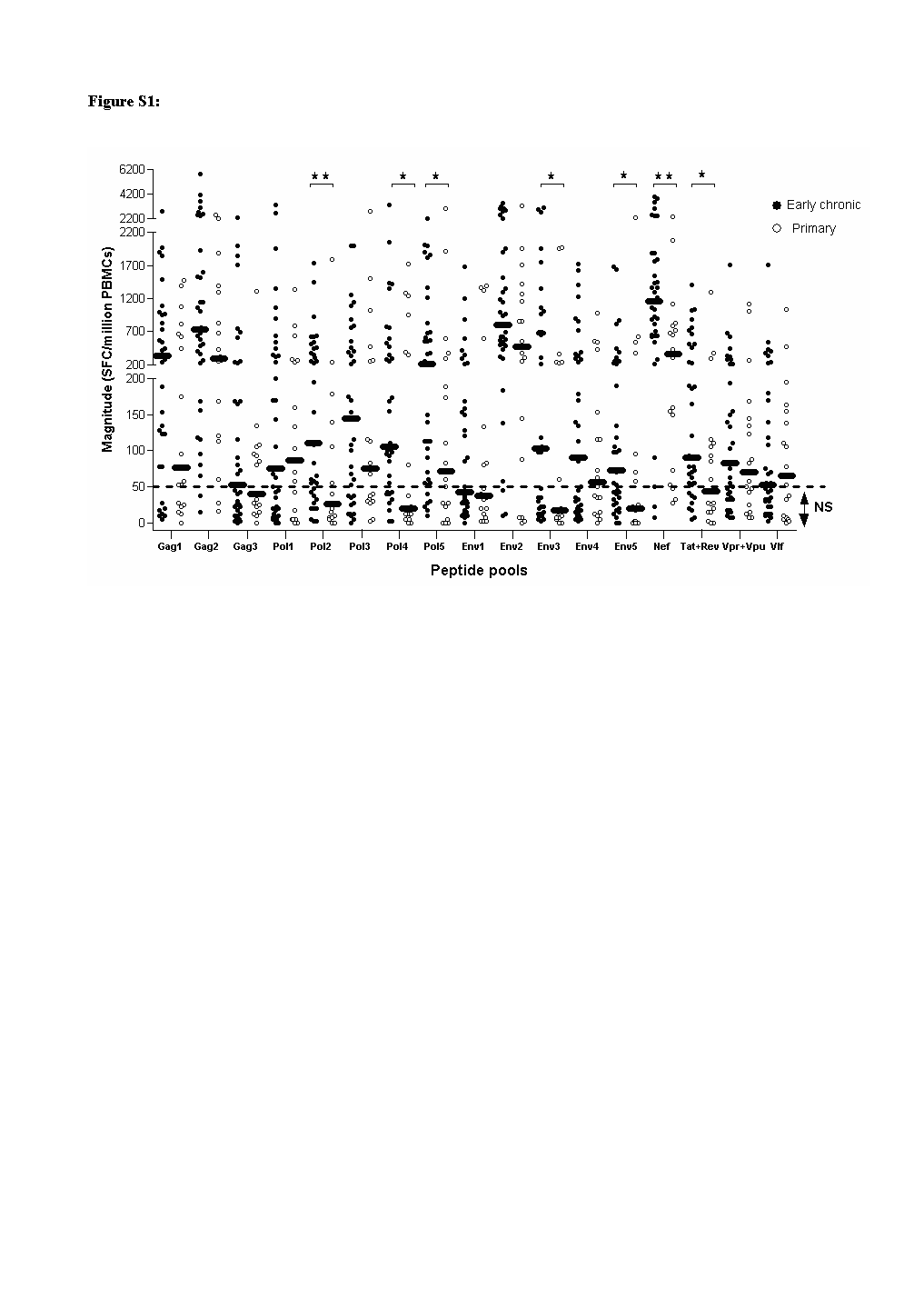

Supplement: Figure S1 — Comparison of the magnitude of CD8+ T cell responses to each peptide pools per individual between groups. Elispot assays were used to determine the magnitude of CD8+ T cell responses. Filled circles were used for early chronic HIV-infected subjects and open circles for primary HIV-infected subjects; The SFCs from control settings were deducted from experimental setting and therefore the spots shown in this figure were “net” SFCs for comparison. Statistically significant differences between groups (p<0.05) were denoted by a line with an asterisk, and p<0.01 for double asterisks. The median magnitude for each group are indicated as horizontal black lines. The p value was calculated by using a nonparametric Mann–Whitney test. (TIFF) [file pone.0044983.s001.tiff]

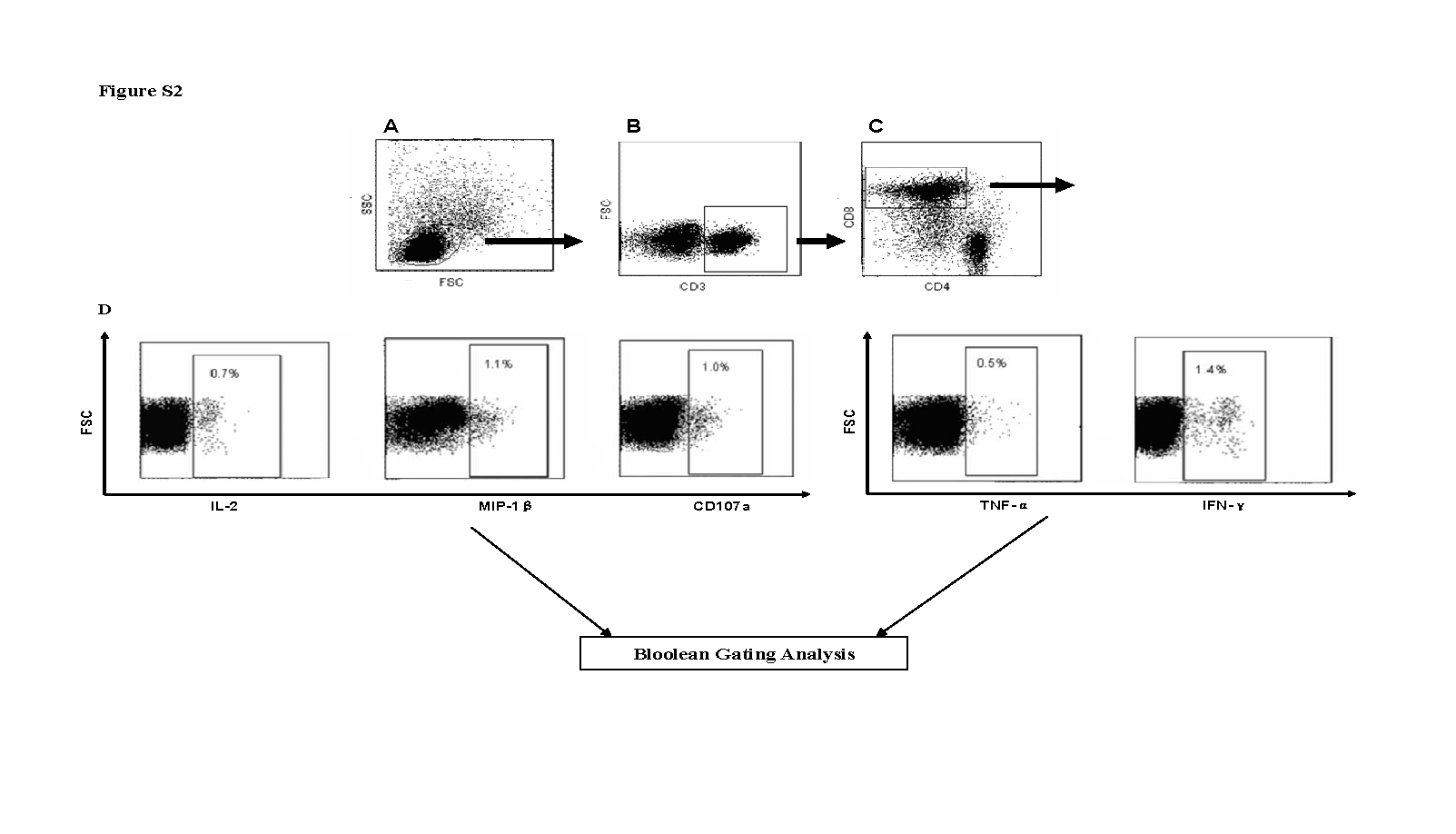

Supplement: Figure S2 — Gating scheme used for the identification of CD8+ T-cell responses. Data shown were from cells derived from one representative patient, stimulated with Gag 1 peptide pool. Initial gating was performed on lymphocytes in a forward scatter of FSC-A versus FSC-H, and then FSC vs SSC plot. CD3+ events were gated in the FSC versus CD3 plot prior to gating on CD3+CD8+ and CD3+CD4+ events. The resulting CD3+CD4-CD8+ population was further gated based on positivity for each of 5 functional responses including IL-2, MIP-1β, CD107a, TNF-α and IFN-γ. (TIFF) [file pone.0044983.s002.tiff]

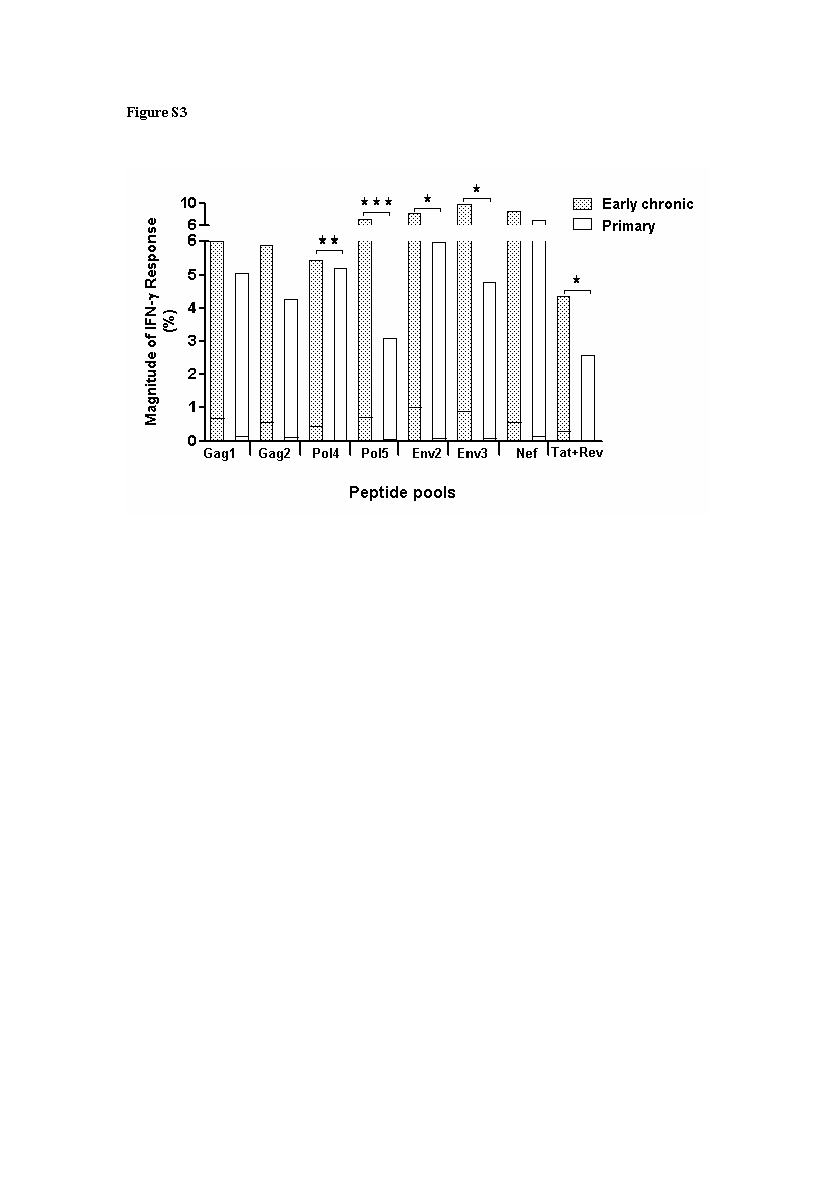

Supplement: Figure S3 — Comparison of the magnitude of CD8+ T cell responses to each peptide pools between groups. Intracellular staining assays were used to determine the magnitude of CD8+ T cell responses. Statistically significant differences between groups (p<0.05) were denoted by a line with an asterisk, and p<0.01 for double asterisks and p<0.001 for three asterisks. The median magnitudes for each group were indicated as horizontal black lines. The p value was calculated by using a nonparametric Mann–Whitney test. (TIFF) [file pone.0044983.s003.tiff]

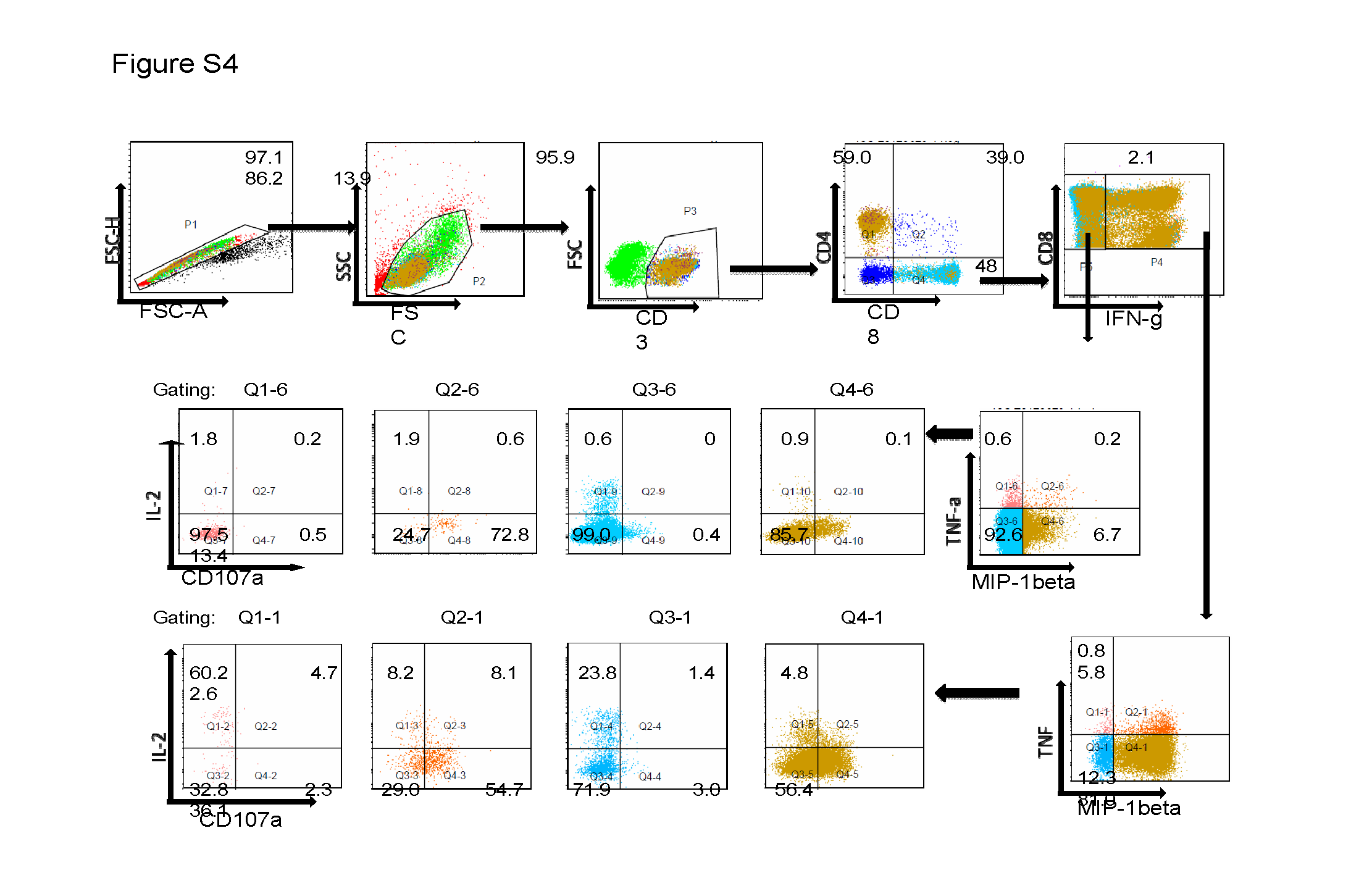

Supplement: Figure S4 — Gating scheme used for the identification of different functional CD8+ T-cell subpopulations. Data shown were from one representative patient, stimulated with PMA plus Ionomycin (positive control). Cells collected from diagonal line in FSC-A vs FSC-H plot were used for subsequent analysis, both CD3 and CD8 surface markers were employed to determine CD8+ T-cell population. Different functional CD8+ T cells were determined as followings: IFN-γ was firstly tested, and then both IFN-γ positive and negative cells were further determined on the plot of TNF-alpha vs MIP-1belta, finally all four populations from the plot of TNF-alpha vs MIP-1belta were tested on the plot of IL-2 vs CD107a. The percentage for each functional CD8+ T cell population was calculated against CD8+ T cells, eg. IFN-γ+TNF-a+MIP-1β+CD107a+IL-2- population is calculated by 13.9%×5.8%×54.6% = 0.44%. (TIFF) [file pone.0044983.s004.tiff]
